# Near-Infrared Spectroscopy of the Urinary Bladder during Voiding in Men with Lower Urinary Tract Symptoms: A Preliminary Study

**DOI:** 10.1155/2013/452857

**Published:** 2013-07-14

**Authors:** Fawzy F. Farag, Joseph Meletiadis, Mohamad D. Saleem, Wout F. Feitz, John P. Heesakkers

**Affiliations:** ^1^Department of Urology, Radboud University Nijmegen Medical Centre, Geert Grooteplein Zuid 10, P.O. Box 9101, Route 659, 6500 HB Nijmegen, The Netherlands; ^2^Department of Urology, Sohag University Hospital, Sohag University, The University Street, Nasser City, 82524 Sohag, Egypt; ^3^Clinical Microbiology Laboratory, Attikon University General Hospital, Medical School, University of Athens, Rimini 1 Street, Haidari 124 62, Athens, Greece

## Abstract

*Objectives*. To determine the difference in response of NIRS of the bladder during voiding between men with and without BOO.LUTS. *Methods*. A prospective, case series, study included 36 men with LUTS. Patients completed the IPSS questionnaire; prostate volumes were measured sonographically. Patients underwent pressure flow study (PFS) with simultaneous NIRS of the bladder. Amplitudes of HHb, O_2_Hb, and Hb_sum_ were calculated at *Q*_max_, relative to baseline. Patients were urodynamically classified as obstructed and unobstructed. Recursive partition analysis (RPA) was performed to reclassify patients using NIRS amplitudes, followed by combined data of NIRS amplitudes, prostate volume, IPSS, and *Q*_max_ to determine the best predictor(s) of BOO. *Results*. PFS classified 28 patients as obstructed and 8 as unobstructed. The median HHb amplitude was significantly higher in obstructed group. RPA of NIRS amplitudes correctly reclassified 89% of patients [AUC: 0.91]. RPA of the combined IPSS, prostate volume, PVR, and *Q*_max_ correctly reclassified 72% of patients [AUC: 0.84]. When NIRS amplitudes were added to this combination, RPA revealed a significantly (*P* < 0.01) higher rate of correct reclassification in 89% of patients with 89.3% sensitivity and 88% specificity for obstruction [AUC: 0.96]. *Conclusion*. NIRS data can be of diagnostic value for BOO in men with LUTS.

## 1. Introduction

Benign prostatic hyperplasia (BPH) is a common disease in elderly men. BPH can be a leading cause of bladder outlet obstruction (BOO) and lower urinary tract symptoms (LUTS). About 25% of men above 60 years require surgical treatment for BOO [1]. BOO is characterized by an increase in detrusor pressure with decreased urinary flow rate during voiding [2]. Pressure flow study (PFS) is the current standard diagnostic test for BOO [3]. However, this test is invasive with potential morbidity. Therefore, it is worthwhile to develop a noninvasive diagnostic technique for BOO.

Doppler ultrasonographic studies and radioisotope labeled microsphere techniques have revealed significant hemodynamic changes in the urinary bladder during the micturition cycle. Near-infrared spectroscopy (NIRS) is a function imaging technology. It enables noninvasive evaluation of oxygen-dependent hemodynamic changes in the biological tissues by measurement of the relevant changes in the concentration of tissue hemoglobin. Oxy-hemoglobin (O_2_Hb) and deoxy-hemoglobin (HHb), respectively, represent oxygen supply and consumption of the tissue. The sum of O_2_Hb and HHb (Hb_sum_) represents the total blood perfusion of the tissue under monitoring [4–6].

Macnab and Stothers [7] developed an algorithm that combined the qualitative NIRS patterns with the maximum flow rate (*Q*_max⁡_) and the postvoid residue (PVR) in the classification of 55 men with LUTS. The authors reported a 85.7% sensitivity and 88.9% specificity of this algorithm for BOO. In a study by Te et al. [8], NIRS algorithm showed a 89% concordance with the conventional urodynamic diagnosis in 36 men with LUTS. Yurt et al. [9] reported 86.2% sensitivity and 87.5% specificity of the NIRS algorithm for BOO. Stothers et al. [10] evaluated 64 men with LUTS using conventional PFS with simultaneous NIRS of the bladder during voiding. Authors reported a good discriminatory ability of NIRS data related to obstruction with 100% sensitivity and 88% specificity for BOO.

In the current study, a quantitative approach of NIRS parameters was used. Our objective was to determine the difference in the response of NIRS data obtained from the bladder wall during voiding between men with and without BOO. A secondary objective was to determine the diagnostic value of NIRS in men with LUTS suggestive of BOO, either alone or in combination with other noninvasive diagnostic parameters of prostate volume, International Prostate Symptom Score (IPSS), and *Q*_max⁡_.

## 2. Patients and Methods

Participants were adult men referred to the Radboud University Nijmegen Medical Centre for urodynamic evaluation of their LUTS. Exclusion criteria were hematuria, a scar of previous pelvic surgery, and a history of radical prostatectomy. The protocol for this study has been approved by local Ethics Committee of Radboud University Nijmegen Medical Centre (2009/188) and conforms to the provisions of the Declaration of Helsinki.

Patients received no medication for their urologic disorders prior to the date of investigation. Patients completed the IPSS questionnaire. Transrectal ultrasound was performed to measure the prostate volume. Dipstick urinalysis was performed to exclude hematuria and urinary tract infection.

All patients underwent PFS (Solar, Medical Measurement Systems, Enschede, The Netherlands). A gas filled urethral catheter (6 Fr/Ch) and a rectal catheter were inserted to monitor intravesical pressure (*P*_ves_) and abdominal pressure (*P*_abd_), respectively. Water was infused at room temperature at a rate of 50 mL/min until maximum capacity was reached. Then, the patients were asked to void in the flowmeter in sitting or standing position according to their preference.

Simultaneous transcutaneous NIRS of the bladder was performed during the PFS (URO-NIRS, Urodynamix Technologies Ltd., Vancouver, BC, Canada). NIRS optodes were placed on the abdomen 2 cm above the pubic symphysis across the midline [11]. Patients were asked to remain still in standing or sitting position to have baseline readings of the NIRS curves. Then, permission to void was given, and patients were asked to avoid straining during voiding. Simultaneous PFS and NIRS data acquisition were performed with a sampling frequency of 10 Hz.

### 2.1. Data Preprocessing and Analysis

Data processing was performed using MATLAB software (MATLAB R2009a, version 7.8.0.374, The Mathworks Inc., Natick, MA, USA). NIRS data was filtered using a second-order Butterworth low-pass filter, with a cut-off frequency of 0.2 Hz.

**Figure 1:**
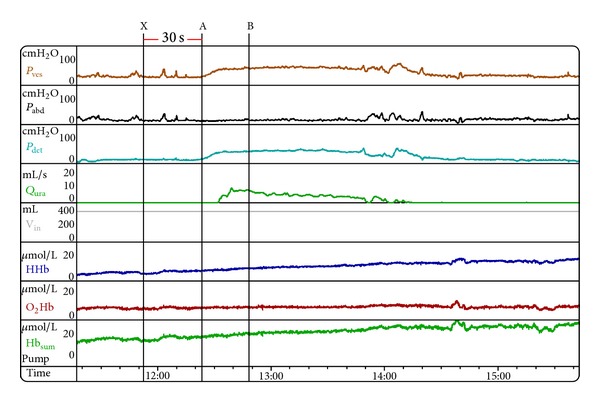
An example of voiding session without potential motion artifacts. Pressure flow study with simultaneous near-infrared spectroscopy (NIRS) of a man with LUTS bladder outlet obstruction. Thirty seconds of baseline was averaged between points “X” and “A” for all NIRS parameters. The amplitudes of change in deoxy-hemoglobin (HHb), oxy-hemoglobin (O_2_Hb), and total hemoglobin (Hb_sum_) were calculated as follows: amplitude of each NIRS parameter = its value at point “B”—the average value of 30 s baseline of that parameter. There is an overall rise in the Hb_sum_ curve led mainly by the relevant rise in the HHb curve which may imply an overall increase in the rate of oxygen consumption during voiding in this subject. *P*_ves_: intravesical pressure, *P*_abd_: abdominal pressure, *P*_det⁡_: detrusor pressure, *Q*_ura_: urinary flow rate.

Two points were marked on detrusor pressure (*P*_det⁡_) curve during voiding activity (Figure 1): point “A” at the beginning of detrusor muscle contraction and point “B” at *P*_det⁡·*Q*_max⁡__. Baseline readings of HHb, O_2_Hb, and Hb_sum_ curves were obtained by averaging 30 seconds of data before point “A.” Relative amplitudes of NIRS variables were calculated by subtracting the averaged 30-second baseline of every variable from point “B” of the same variable.

Patients were Urodynamically classified as obstructed or unobstructed using “detrusor/flow plot” according to Griffiths et al. [12]. Diagnosis of obstruction was made when group-specific resistance factor (URA) was >28. To obtain an 80% probability of detecting a difference of 3 *μ*mol/L in NIRS amplitudes between the obstructed and the unobstructed groups, a 2-tailed alpha level was set at *α* = 0.05 and a standard deviation of 3; the sample size needed was found to be 33 patients.

Median (range) demographic, urodynamic, and NIRS parameters were calculated. The Mann Whitney *U* test was applied to test the differences between groups. Differences with *P* values less than 0.05 were considered statistically significant.

Recursive partition analysis (RPA) [13] was then performed to test the ability of NIRS relative amplitudes to predict obstruction. RPA is a nonparametric method that recursively partitions data for relating independent variable(s) (NIRS and other noninvasive parameters) to a dependent variable (urodynamic diagnosis) creating a tree of partitions. It finds a set of cuts of the independent variable(s) that best predict the dependent variable, by searching all possible cuts until the desired fit is reached. RPA was performed to test the ability of the combined data of prostate volume, IPSS, and *Q*_max⁡_ to predict obstruction. Finally, RPA of the combined data of NIRS amplitudes, *Q*_max⁡_, IPSS, and prostate volume was performed to explore the clinical usefulness of quantitative NIRS when combined with these parameters in the diagnosis of BOO in men with LUTS.

The sensitivity and specificity in predicting obstruction together with the area under the receiver-operating curve (AUC) were calculated. An AUC of 1 indicates a perfect test in predicting obstruction. The % of correct classifications obtained with the three different RPA were compared using Students *t*-test for proportions based on their SDs calculated as √(*p*∗(1 − *p*)/*N*), where *p* is the proportion and *N* the sample size.

Statistical analysis was done with MATLAB (The Mathworks Inc., Natick, MA, USA) and JMP 7.0.2 (SAS Institute, Cary, NC, USA).

## 3. Results

Thirty-six men with LUTS were included in this study. The mean age was 67 ± 9.3 y (range: 42–86), mean BMI was 26.6 ± 3.2 kg/m^2^ (range: 21–34), mean IPSS was 17 ± 7 (range: 5–33), and mean prostate volume was 52.6 ± 33 cm^3^ (range: 21–160).

**Figure 2:**
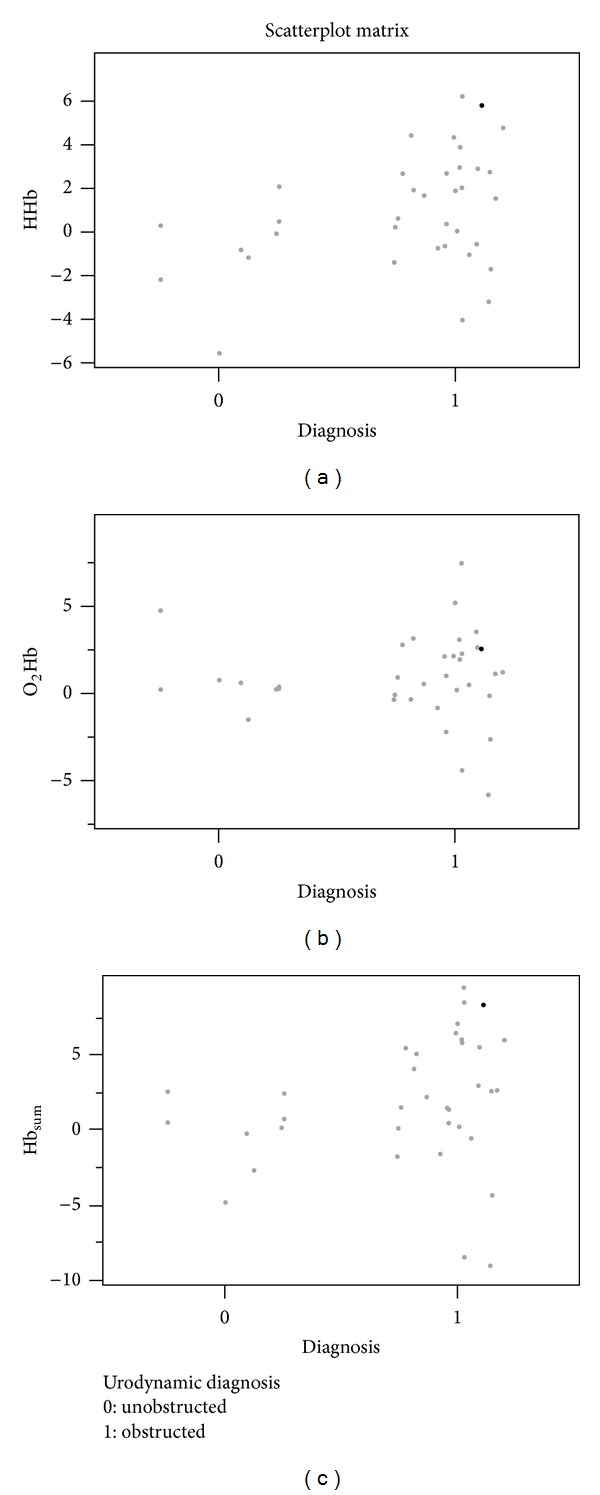
Scatterplot amplitudes of deoxy-hemoglobin (HHb), oxy-hemoglobin (O_2_Hb), and total hemoglobin (Hb_sum_) based on presence or absence of obstruction according to standard urodynamic diagnosis. Despite the overlapping, NIRS parameters were significantly higher in obstructed patients compared to unobstructed patients.

Twenty-eight patients (77%) had urodynamic obstruction, while eight patients (23%) were unobstructed. The median relative amplitudes of HHb were significantly higher in obstructed group (*P* = 0.045).  Table 1 shows a comparison of NIRS, urodynamic, and other relevant parameters between obstructed and unobstructed groups.  Figure 2 shows results of scatterplot NIRS amplitudes of HHb, O_2_Hb, and Hb_sum_ based on presence or absence of obstruction.

Four of 36 patients had overt abdominal activity during voiding represented by exaggerated rise in the NIRS parameters during voiding in these subjects. Although this rising implies increased oxygen consumption or muscle fatigue during voiding in these patients, we tried to minimize any potential artifact in NIRS. Therefore, analysis was repeated after exclusion of these 4 patients. The median relative amplitude of HHb was still higher in the obstructed group (1.0 *μ*mol/L, *n* = 25) than the unobstructed group (−1.0 *μ*mol/L, *n* = 7), but without statistical significance (*P* = 0.12).

**Figure 3:**
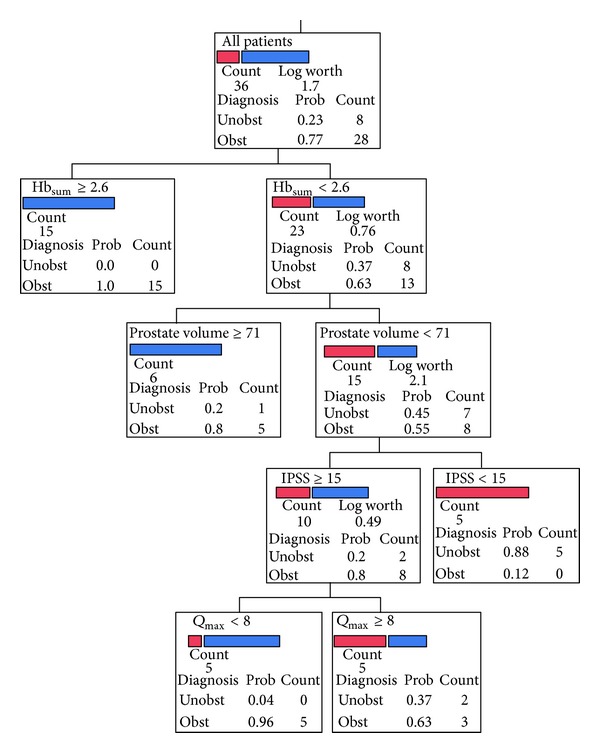
Classification responses of recursive partition analysis of the combined noninvasive parameters of near-infrared spectroscopy (NIRS), prostate volume, maximum flow rate (*Q*_max⁡_), and International Prostate Symptom Score (IPSS). −log⁡_10_ (*P* value); prob: probability of having the diagnosis either obstructed or unobstructed. The total number of patients is 36. The number of true positives is 25 of 28, the number of false positives is 1 of 8, the number of true negatives is 7 of 8, and the number of false negatives is 3 of 28. The sensitivity and specificity for obstruction are 89.3% and 88%, respectively, [AUC: 0.96]. It is to be mentioned that these statistical values need to be tested in a new set of patients before they can be considered for clinical application.

RPA of NIRS relative amplitudes revealed that NIRS correctly classified patients as obstructed or unobstructed in 32 of 36 patients (89%) with 96% sensitivity and 62% specificity, 90% PPV, and 62% NPV for BOO [AUC: 0.92]. RPA of the combined *Q*_max⁡_, IPSS, PVR, and prostate volume correctly classified 26 of 36 patients (72%) with 71% sensitivity and 75% specificity, 91% PPV, and 75% NPV for BOO [AUC: 0.84]. When NIRS amplitudes were added to this combination, RPA showed correct classification in 32 of 36 patients (89%), with 89.3% sensitivity and 87.5% specificity, 96.3% PPV, and 87.5% NPV for BOO [AUC: 0.96]. This increase of % correct classifications was statistically significant (*P* < 0.01).  Figure 3 shows the classification responses of this noninvasive combination; the computed probability of having the diagnosis “obstructed” was 100% using the cut-off Hb_sum_ ≥ 2.6 *μ*mol/L in 15 of 36 patients. For patients with Hb_sum_ amplitudes <2.6 *μ*mol/L, the prostate volume was checked; a cut-off value ≥ 71 grams had a 100% probability to be obstructed in five of 36 patients. For patients with prostate volume < 71 grams, the IPSS was checked; an IPSS < 15 had a 0% probability of being obstructed in five of 36 patients. For patients with IPSS ≥15, the *Q*_max⁡_ was checked; *Q*_max⁡_ cut-off value <8 mL/s had an 100% probability of being obstructed in 5 of 36 patients while a *Q*_max⁡_ ≥ 8 mL/s had a 62% probability of being obstructed in 3 of 36 patients.

## 4. Discussion

Hemodynamic studies of the skeletal muscles revealed that forceful contraction results in compression of the muscle vasculature with expulsion of the blood into the extra-muscular venous compartment “muscle pump” effect [14]. Perhaps the same could be true for the detrusor muscle contraction during voiding, and moreover an even higher venous outflow could be expected in subjects with BOO due to the more powerful detrusor muscle contraction to overcome the high bladder outlet resistance in these subjects. However, it would be very interesting to test if this applies also to the severely dysfunctional detrusor with compromised contractile force.

Specific hemodynamic studies of the bladder wall showed a spectrum of inconsistent results. Azadzoi et al. [15] observed a decrease in blood flow to the bladder wall during spontaneous and evoked contractions in animal studies. Kershen et al. [16] showed that the urinary blood flow is increased with the increase in bladder pressure and volume, while Kroyer et al. [17] reported inconsistent variations in mucosal and muscular blood flow of the bladder with increased bladder pressure in dogs. In their experiment, the blood flow of the bladder was increased in one dog, decreased in another dog, and showed no change in a third dog.

Qualitative NIRS has been applied in the monitoring of the bladder hemodynamics during the voiding phase [7, 10, 11] and the filling phase [18] of the micturition cycle. In the current study, relative changes in the concentration of hemoglobin in the bladder wall were quantified during the initial phase of detrusor muscle contraction until the point of *Q*_max⁡_. We believe that this part of the voiding act is the most active phase of the voiding cycle with substantial hemodynamic changes being expected due to central and/or peripheral regulatory mechanisms of the blood flow to the bladder wall and the mechanical effect of detrusor muscle contraction on the bladder wall vasculature (myogenic effect).

NIRS measures changes in the hemoglobin concentration and the oxygen consumption in biological tissues, mainly from the venous blood compartment [19–21]. In the current study, the median amplitude of change in HHb was significantly higher in the patients group with BOO. This overall trend may reflect a pathophysiologic phenomenon of increased oxygen extraction during more powerful detrusor muscle contraction which can be explained in the light of the “muscle pump” phenomenon (Figure 1). However, it has to be mentioned that this pattern could also reflect part of a wider spectrum of the detrusor hemodynamic changes in subjects with LUTS due to BOO that ranges from minimal effect to frank ischemic changes in some subjects.

Doppler ultrasound studies have shown a high arterial resistive index in patients with BOO [22–24]. These studies attributed their findings to potential ischemic changes in the bladder wall due to BOO. We think that the time factor and the degree of chronicity of BOO that may lead to the development of this proposed ischemia should be taken into account, meaning that it may not occur in patients with recent history of LUTS/BOO. Our findings may explain the inconsistency in NIRS patterns reported by Chung et al. [25]. The authors applied a noninvasive diagnostic algorithm of *Q*_max⁡_, PVR, and qualitative NIRS patterns during voiding in reclassification of men with LUTS. This algorithm came with low diagnostic value for BOO [AUC: 0.48], and interestingly 35% of patients with BOO had a downward, 15% had a flat, and 50% had an upward NIRS pattern, while 57% of patients with no BOO had a downward, 14% had a flat, and 29% of these patients had an upward NIRS pattern. The inconsistency in NIRS patterns in Chung's study might reflect a spectrum of hemodynamic changes representing pathophysiologic variations in the detrusor muscles of subjects with BOO.

An exploratory analysis was further performed to explore potential usefulness of NIRS in urological clinical practice. Results of RPA using the relative amplitudes of NIRS variables revealed their ability to correctly classify 89% of patients as having BOO or no BOO, while classifying the patients using prostate volume, IPSS, PVR, and *Q*_max⁡_ was correct in only 72% of the patients. This indicates that NIRS data would be of value in the diagnosis of BOO independently. When NIRS data were combined with other parameters like prostate volume, IPSS, PVR, and *Q*_max⁡_, it significantly improved their diagnostic performance to predict BOO.

A classification model was developed combining all these parameters; the software found no suitable cuts in the PVR parameter that matches the desired fit, and therefore PVR was automatically excluded from this model. This model successfully classified 89% of patients with 89.3% sensitivity and 87.5% specificity, 96.3% PPV, and 87.5% NPV for BOO [AUC: 0.96]. In previous work by Stothers et al. [10], the authors developed a classification and regression tree model using relative changes in the concentration of HHb and O_2_Hb from the start to the end points of the urinary outflow with high sensitivity (100%) and specificity (89%) of NIRS to BOO. The mathematical model applied in this study is fully described by Guevara et al. [26].

Abdominal straining can lead to exaggerated NIRS response [27]. Four patients were excluded due to overt abdominal activity during voiding. The median relative amplitude of HHb was still higher in the obstructed group, but with no statistical significance, which still implies a potential increase in the oxygen consumption during voiding, and therefore such data can still be of physiologic value even in the presence of abdominal activities with potential motion artifacts. For optimal application of NIRS in clinical practice, we recommend a strict instruction to the patients to avoid using abdominal straining during the measurements. Finally, algorithms to cancel motion artifacts in NIRS are currently available [28]; it will be of great value to develop a specific algorithm for NIRS applications in urology.

A limitation of our study would be the difficulty to select the time window within which the NIRS changes were quantified, especially for the future studies when the NIRS will be used alone without a reference test like the PFS. A solution would be to apply point “A” as the point of first deviation from a stable baseline before the onset of the urinary flow. Another limitation would be the relatively small sample size to run a RPA in our data set. Another important observation on the current data is that, in spite of the overall trend of HHb variable of the NIRS data to be higher in the obstructed group, there was some overlap between the obstructed and unobstructed groups as shown in Figure 2. Therefore, the algorithm developed in the current study needs to be validated in larger studies. One last point to clarify is that the *Q*_max⁡_ values used in the algorithm were obtained from the PFS and not from the free flow test of the patients. In the future application of this algorithm, including the *Q*_max⁡_ values obtained from the free flow tests of the patients should be considered to establish the noninvasive diagnosis in men with LUTS.

## 5. Conclusions

When applying NIRS in men with LUTS during voiding, there is an overall trend of the relative median amplitude of HHb variable to be higher in men with BOO. This seems to be of physiologic origin due to increased amount of oxygen consumed during voiding. NIRS data can be of diagnostic value in men with LUTS. An algorithm is being developed in our series that would provide hemodynamic, anatomical, and functional evaluation of the lower urinary tract. Moreover, it esteems the impact of BOO on the patient.

## Figures and Tables

**Table 1:** Comparison of demographic, urodynamic, and near-infrared spectroscopy (NIRS) parameters between the obstructed and unobstructed groups.

Statistics (median)	Obstructed *n* = 28	Unobstructed *n* = 8	*P* value*
Age (yr)	68 (52–86)	68.5 (42–76)	0.99
BMI (Kg/m^2^)	26.5 (20–32)	26.6 (24–34)	0.69
IPSS	19 (7–28)	12 (5–33)	0.23
Prostate volume	42 (23–160)	32 (21–50)	0.15
Voided volumes	172 (16–600)	201 (97–424)	0.69
*Q*_max⁡_ (mL/s)	5 (2–12)	7 (2–9)	
*P*_det⁡·*Q*_max⁡__ (cmH_2_O)	69 (37–175)	39 (26–47)	
PVR (mL)	169 (0–682)	103 (0–350)	0.69
HHb (*µ*mol/L)	1.8 (−4.0–6.2)	−4.5 (−5.6–2.1)	0.045*
O_2_Hb (*µ*mol/L)	1.1 (−5.8–7.5)	0.32 (−1.5–4.8)	0.09
Hb_sum_ (*µ*mol/L)	2.6 (−9.0–9.5)	0.33 (−4.8–2.6)	0.23

BMI: body mass index, IPSS: International Prostate Symptom Score, PSA: prostate specific antigen, *Q*_max⁡_: maximum flow rate, PVR: postvoid residual urine, HHb: deoxy-hemoglobin, O_2_Hb: oxy-hemoglobin, Hb_sum_: total hemoglobin (Hb_sum_ = HHb + O_2_Hb), *P*_det⁡·*Q*_max⁡__: detrusor pressure at maximum flow rate.

*P value <0.05 is significant (Mann Whitney *U* test).
